# Atlanta Medical College

**Published:** 1877-04

**Authors:** 


Atlanta Medical College.—The annual commencement of this institution was held on the first day of March, after a course of four and a-half months. The degree of Doctor in Medicine, was conferred on twenty-two applicants, by Col. Wm. L. Mitchell, one of the Trustees, appointed by the Board for this service.

The prize, a case of surgical instruments, offered by the Faculty for the greatest proficiency, as exhibited in the general examination, was awarded to J. W. Williams, of Missouri. He was the fortunate recipient also of the ophthalmoscope offered by the Professor of Diseases of the Eye and Ear, for the best essay on this branch.

The presentation of prizes was made by Gen. W. S. Walker, preceeded by an appropriate address to the graduates.

Prof. Wm. Abram Love delivered the valedictory address on the part of the Faculty, and J. W. Williams on the part of the class.

The following names form the catalogue of graduates for the session of 1876–7:

Adams, Wm. Edgar, Greensboro, Ga.
Ansley, J. S., Pigeon Creek, Ala.
Boyd, Chas., Atlanta, Ga.
Brown, G. W., Brownsville, Ala.
Bullard, W. L., Tennille, Ga.
Cook, R. A., Covington, Ga.
Findlay, C. D., Twiggsville, Ga.
Goldin, W. F., Buchanan, Ga.
Greer, L. C., Beaverdale, Ga.
Haynes, Micajah, Red Clay, Ga.
Howell, D. H., Atlanta, Ga.
Jackson, J. T., Thomaston, Ga.
Martin. J. G., Milner, Ga.
Perry, C. F., LaFayette, Ala.
Smith, H. M., Newnan, Ga.
Smith, J. Horace, Smyrna, Ga.
Spears, A. F., Madison, Ga.
Statham, J. P., Long Cane, Ga.
Ticknor, D. C., Columbus, Ga.
Walker, G. T., Greeneville, S. C.
Weaver, J. L., Cochran, Ga.
Williams, J. W., Farmington, Mo.

